# Zero‐inflated count distributions for capture–mark–reencounter data

**DOI:** 10.1002/ece3.9274

**Published:** 2022-09-09

**Authors:** Thomas V. Riecke, Daniel Gibson, James S. Sedinger, Michael Schaub

**Affiliations:** ^1^ Swiss Ornithological Institute Sempach Switzerland; ^2^ Warner College of Natural Resources Colorado State University Fort Collins Colorado USA; ^3^ Department of Natural Resources and Environmental Science University of Nevada Reno Nevada USA

**Keywords:** Bayesian, capture–mark–recapture, gamma‐Poisson, individual heterogeneity, mark‐resight, robust design, temporary emigration, zero‐inflation

## Abstract

The estimation of demographic parameters is a key component of evolutionary demography and conservation biology. Capture–mark–recapture methods have served as a fundamental tool for estimating demographic parameters. The accurate estimation of demographic parameters in capture–mark–recapture studies depends on accurate modeling of the observation process. Classic capture–mark–recapture models typically model the observation process as a Bernoulli or categorical trial with detection probability conditional on a marked individual's availability for detection (e.g., alive, or alive and present in a study area). Alternatives to this approach are underused, but may have great utility in capture–recapture studies. In this paper, we explore a simple concept: *in the same way that counts contain more information about abundance than simple detection/non‐detection data, the number of encounters of individuals during observation occasions contains more information about the observation process than detection/non‐detection data for individuals during the same occasion*. Rather than using Bernoulli or categorical distributions to estimate detection probability, we demonstrate the application of zero‐inflated Poisson and gamma‐Poisson distributions. The use of count distributions allows for inference on availability for encounter, as well as a wide variety of parameterizations for heterogeneity in the observation process. We demonstrate that this approach can accurately recover demographic and observation parameters in the presence of individual heterogeneity in detection probability and discuss some potential future extensions of this method.

## Graphical abstract

In this paper we explore a simple concept: in the same way that counts provide more information about abundance than detection/non‐detection data, counts of the number of observations of uniquely marked individuals can provide more information about demographic parameters than detection/non‐detection data. Zero‐inflated parameterizations of capture–recapture models can decrease runtime, and improve the estimation of heterogeneity in detection probability among individuals.

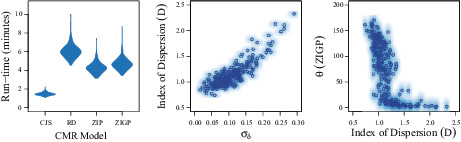

## 1 INTRODUCTION

The estimation of demographic parameters is fundamental to successful conservation and evolutionary ecology. Since their initial development, capture–mark–recapture (hereafter, CMR) models have been used to estimate demographic parameters such as apparent survival (Cormack, 1964; Jolly, 1965; Seber, 1965), true survival and site fidelity (Burnham, 1993), transitions among discrete strata (Brownie et al., 1993), temporary emigration or breeding probability (Kendall et al., 1995, 1997), recruitment (Pradel, 1996), and the spatial distribution of organisms (Royle et al., 2013; Royle & Young, 2008). Parameter estimates from CMR models are often used as vital components of population models (Caswell, 2000; Schaub & Kéry, 2021) and to develop a more complete understanding of individual fitness (Cam et al., 2002; Stearns, 1992). CMR models typically consist of two primary components: (1) a model of latent biological processes (i.e., survival, movement among populations, emigration, disease dynamics), and (2) a model of the observation of uniquely identifiable individuals. Models of both latent biological and observation processes typically take the form of categorical or Bernoulli distributions, and individuals are grouped into discrete groups or states (e.g., alive or dead, observed or not observed).

Heterogeneity among “uniquely identifiable” (hereafter, marked) organisms in both biological processes (e.g., Cam et al., 2002; Pledger & Schwarz, 2002) and observation probability (e.g., Pledger, 2005; Pollock, 1982) has long been recognized as a central challenge in CMR modeling (Otis et al., 1978). In a seminal paper, Pollock (1982) proposed that heterogeneity in detection might be accounted for by subdividing primary occasions into multiple secondary occasions. Similarly, Fletcher (1994) developed a method for modeling the probability of encounter of individuals as a function of the number of unique resights of that individual during the previous occasion. Shortly thereafter, Kendall and others (Kendall et al., 1995, 1997) expanded the method developed by Pollock (1982) to estimate availability for encounter (i.e., zero‐inflation) by partitioning primary occasions into shorter secondary occasions, assuming closure among secondary occasions within a primary occasion, and estimating probabilities of temporary emigration from the study area. Since that time, methods have been developed to estimate individual detection probabilities using random effects (Clark et al., 2005; Royle & Dorazio, 2008) or mixtures (Pledger, 2000; Pledger et al., 2003). More recent efforts have simultaneously used information about marked organism location and the locations of sampling efforts to model spatial variation in reencounter probability (Royle et al., 2013; Royle & Young, 2008). However, the estimation of heterogeneity in the observation process remains a key challenge in CMR studies, and the continued development of alternative approaches is critical for improved parameter estimation.

Heterogeneity in the detection of marked organisms is often driven by two primary processes. The first is whether or not an individual is even present within the bounds of the study area (i.e., temporary emigration as a source of zero‐inflation; Kendall et al., 1995; Schaub et al., 2004). The second is variation among the latent encounter probabilities of individuals that are present. This latent heterogeneity can be affected by factors such as variation in individual behavior, life stage, and location relative to sampling effort (Royle & Young, 2008). When primary occasions extend over multiple days, weeks, or months, this can lead to some individuals being encountered many times while others are rarely, if ever, detected. The key concept in this paper is that *in the same way that counts contain more information about the abundance of a population than simple detection/non‐detection data, the number of encounters of marked individuals may contain more information about the observation process than detection/non‐detection data* (e.g., McClintock et al., 2009, 2019; McClintock & White, 2009). Thus, rather than summarizing capture–reencounter data using ones (encountered) and zeroes (not encountered) during a primary occasion or multiple secondary occasions, capture–reencounter data can also be summarized as counts of the number of times each marked individual was encountered during a primary occasion (McClintock et al., 2019; McClintock & White, 2009). The number of encounters can then be modeled using a variety of discrete distributions, such as the Poisson or negative binomial distributions. If model assumptions are met, this approach provides a flexible and useful approach to modeling the observation process and may improve upon existing tools to estimate heterogeneity in encounter probability among individuals. Notably, improved estimates of heterogeneity in the observation process lead to improved estimates of demographic parameters. In this paper, we (1) demonstrate the use of this approach with simulated data, (2) describe potential benefits relative to more traditional approaches, (3) demonstrate several approaches for modeling individual heterogeneity in encounter probability, and (4) discuss possible future extensions and uses of this parameterization.

## 2 METHODS

We simulated 250 CMR datasets, each with 10 primary occasions (T=10). For each simulation, we released 25 marked individuals in the first through ninth primary occasions, for a total of 225 released individuals (I=225). We simulated the latent state of each individual ($z_{i,t}$; 1: alive, 0: dead) from occasion to occasion as, $z_{i,t}∼\text{Bernoulli}\left(z_{i,t-1}\phi \right)$, given a survival probability generated from a beta distribution, $\phi ∼\text{beta}\left(40,10\right)$. If an individual was alive in occasion *t*, we simulated its availability for encounter ($a_{i,t}$; 1: available, 0: unavailable) given simulated Markovian (Kendall et al., 1997) probabilities of availability for encounter (γ),

(1) ai,t∼Bernoullizi,t×γ1×1−ai,t−1+zi,t×γ2×ai,t−1,γ1∼beta10,20,γ2∼beta20,10.

These probabilities are directly analogous to parameters described by Kendall et al. (2013), such that $\gamma_{2}$ in this study is equal to the probability of availability given availability in t−1, or $a^{\prime \prime }$ as defined by Kendall et al. (2013), and $\gamma_{1}$ in this study is equal to the probability of availability given absence in t−1, or $a^{\prime }$ as defined by Kendall et al. (2013). During each primary occasion, we sampled individuals that were available for detection for 21 consecutive days (J=21, that is, 3 weeks) given simulated individual random variation in daily detection probability ($d_{i}$; Dorazio et al., 2013; Gomez et al., 2018). Thus, the simulated capture–recapture data form a 3‐dimensional array (*Y*) with dimensions I×T×J,

(2) yi,t,j∼Bernoulliai,t×di,di∼betaμδ×1σδ21−μδ×1σδ2,μδ∼beta10,90,σδ∼gamma5,50,

where $\mu_{\delta }$ is the simulated mean daily detection probability of an average individual, and $\sigma_{\delta }$ is the amount of among‐individual heterogeneity in detection. We then summarized the daily CMR data for analysis with four different model types: (1) a Cormack–Jolly–Seber model where the secondary captures are ignored (CJS; Cormack, 1964; Jolly, 1965; Seber, 1965), (2) a robust design model (RD; Kendall et al., 1995, 1997), and two capture–recapture models with count‐based observation likelihoods, (3) a zero‐inflated Poisson (ZIP), and (4) a zero‐inflated gamma‐Poisson with heterogeneity in the number of encounters per individual (ZIGP). To summarize the CMR data (*M*) for a CJS model, we constructed an I×T matrix and filled the matrix as a function of whether or not an individual was observed on any day during a primary occasion,

(3) $$m_{i,t}∼\left\{\begin{array}{ll} 1, & \text{if}\sum_{j=1}^{21}y_{i,t,j}\geq 1\\ 0, & \text{otherwise} \end{array}\right..$$

To summarize the robust design encounter data (*R*) for the robust design capture–reencounter model, we subdivided each 21‐day long primary occasion into three one‐week long secondary occasions (K=3). If an individual was observed on any day of a week in a secondary occasion, then that secondary occasion ($r_{i,t,k}$) equaled one. If an individual was not observed on any day during a specific secondary occasion, then $r_{i,t,k}=0$. Finally, we summarized the counts of reencounters by individual and primary occasion by simply summing the total number of encounters of each individual during each primary occasion, $c_{i,t}=\sum_{j=1}^{21}y_{i,t,j}$.

In the same way that the data were generated, all four capture–recapture models share a common likelihood for the survival process. The latent state of each individual during each occasion ($z_{i,t}$) was modeled as a function of the individual's latent state in the previous occasion ($z_{i,t-1}$) and a survival probability (ϕ), $z_{i,t}∼\text{Bernoulli}\left(z_{i,t-1}\times \phi \right)$. A vague prior was used for survival, $\phi ∼\text{beta}\left(1,1\right)$. For the CJS model, we then simply modeled the primary occasions encounter data (*M*) as a function of the individual's latent state and a detection probability (*p*), $m_{i,t}∼\text{Bernoulli}\left(z_{i,t}\times p\right)$. We specified a vague prior for detection probability $p∼\text{Beta}\left(1,1\right)$. For the remaining three models, we also estimated whether an individual was available for detection ($a_{i,t}$) given its previous state ($a_{i,t-1}$) and vague priors for Markovian probabilities of availability for encounter (γ; Kendall et al., 1997).

(4) ai,t∼Bernoullizi,t×γ1×1−ai,t−1+zi,t×γ2×ai,t−1,γ1∼beta1,1,γ2∼beta1,1.

For the robust design model, we modeled whether or not each individual was detected during each secondary occasion as a function of its latent availability for detection during the primary occasion ($a_{i,t}$) and a secondary occasion detection probability (*p*). We then derived primary occasion detection probability (p*) from the secondary occasion detection probabilities for comparison of parameter estimates among models,

(5) ri,t,j∼Bernoulliai,t×p,p∼Beta1,1,p*=1−1−p3.

For the zero‐inflated Poisson model, we model the total number of encounters of each individual during each primary occasion ($c_{i,t}$) given availability for detection ($a_{i,t}$) an expected mean number of encounters per individual per primary occasion (ϵ),

(6) ci,t∼Poissonai,t×ϵ,ϵ∼Gamma1,1.

For the zero‐inflated Gamma‐Poisson model with heterogeneity in the number of expected observations per individual, we modeled the number of encounters of each individual during each primary occasion ($c_{i,t}$) given availability for detection ($a_{i,t}$), the mean expected number of encounters per individual (ϵ), and individual encounter heterogeneity ($h_{i}$) estimated using an overdispersion parameter (θ),

(7) ci,t∼Poissonai,t×ϵ×hi,ϵ∼gamma1,1,hi∼gammaθθ,θ∼uniform0,250.

This parameterization is similar to Gamma‐Poisson formulations of the negative binomial distribution (Greene, 2008); however, here we assume heterogeneity among individuals, not observations. Fitting these models in a Bayesian framework allows users to easily customize existing described count distributions for use in these model types. We called JAGS (Plummer, 2003) from R (R Core Team, 2018) using the jagsUI package (Kellner, 2016). For each simulated dataset, we sampled three MCMC chains of 50,000 iterations with an adaptive phase of 1000 iterations. We discarded the first 10,000 iterations and retained every tenth saved iteration. We assessed convergence visually, and chains converged acceptably. We calculated mean signed difference (MSD) as the mean of the differences between the median of the posterior distribution and the true parameter value used to simulate the data, and we calculated coverage as the proportion of simulations in which the 95% symmetric credible intervals included the true parameter value used to simulate the data.

## 3 RESULTS

**FIGURE 1:**
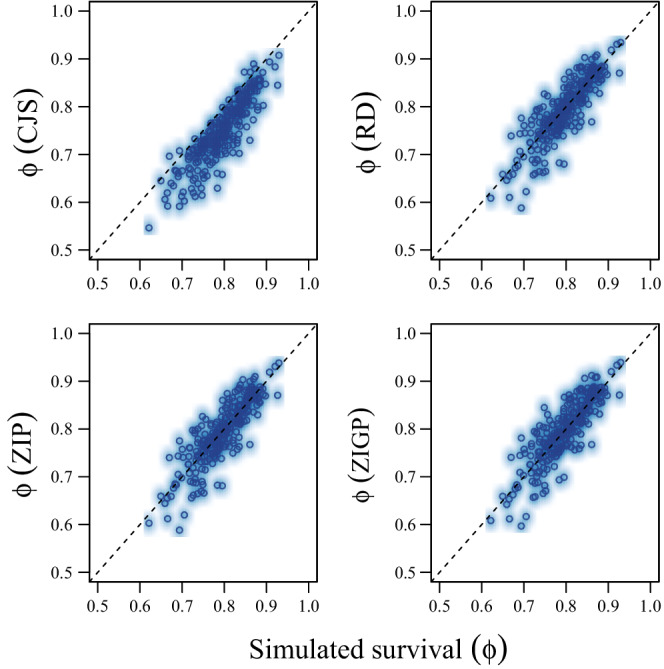
Scatter and density plots of the medians of posterior distributions for apparent survival relative to truth (ϕ) from Cormack–Jolly–Seber (CJS; upper left), robust design (RD; upper right), zero‐inflated Poisson (ZIP, lower left), and zero‐inflated gamma‐Poisson with individual heterogeneity (ZIGP; lower right), capture–mark–reencounter models used to analyze 250 simulated capture–mark–reencounter datasets.

**FIGURE 2:**
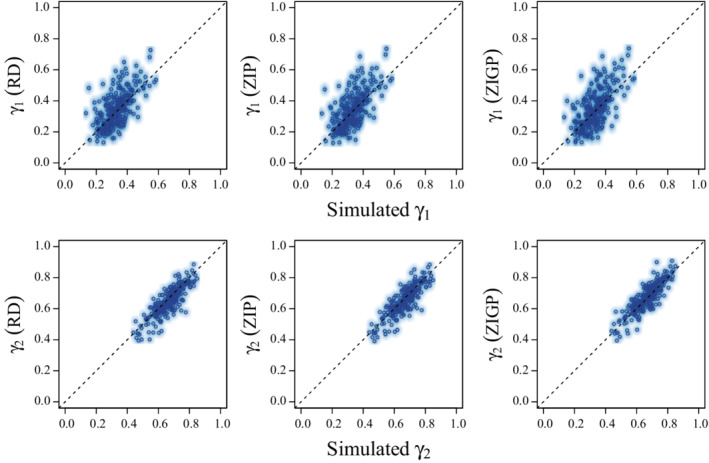
Scatter and density plots of the medians of posterior distributions for availability for encounter relative to truth (γ) from robust design (RD; left), zero‐inflated Poisson (ZIP, center), and zero‐inflated gamma‐Poisson with individual heterogeneity (ZIGP; right), capture–mark–reencounter models used to analyze 250 simulated capture–mark–reencounter datasets.

**FIGURE 3:**
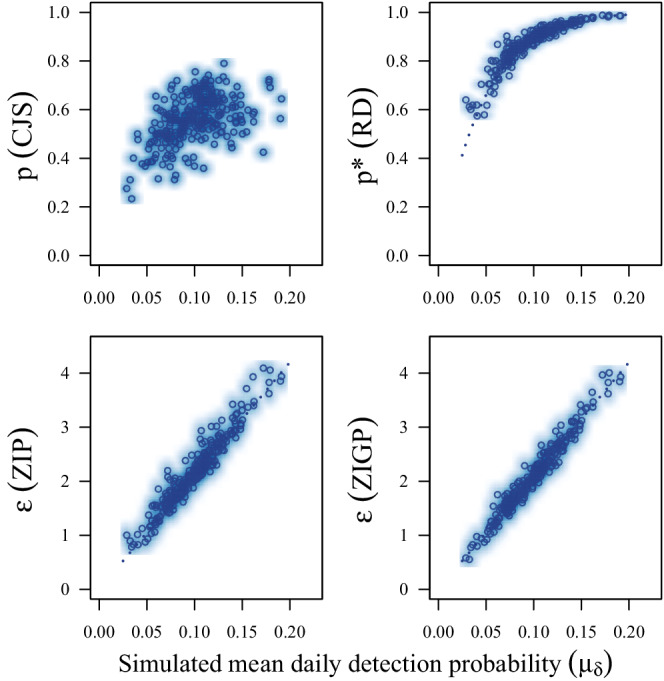
Scatter and density plots of the medians of posterior distributions for primary occasion detection probability (*p*) or the expected number of encounters per individual (ϵ) from Cormack–Jolly–Seber (CJS; upper left), robust design (RD; upper right), zero‐inflated Poisson (ZIP, lower left), and zero‐inflated Poisson with individual heterogeneity (ZIGP; lower right), capture–mark–reencounter models used to analyze 250 simulated capture–mark–reencounter datasets.

**FIGURE 4:**
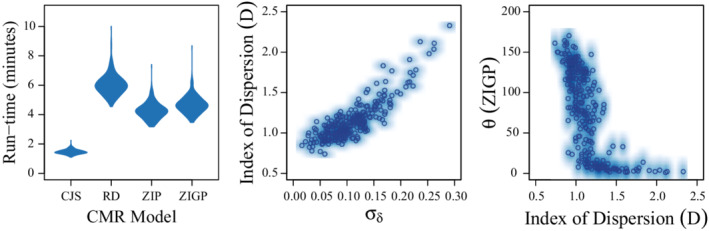
Violin plots of model run times across 250 simulations for Cormack–Jolly–Seber (CJS; Cormack, 1964; Jolly, 1965; Seber, 1965), robust design (RD; Kendall et al., 1995, 1997), zero‐inflated Poisson (ZIP; this study) and zero‐inflated gamma‐Poisson (ZIGP; this study) capture–mark–recapture models (left), scatter plots of the index of dispersion (*D*; Var(*C*)/Mean(*C*)) for the capture–mark–reencounter count data relative to the simulated heterogeneity in detection probability among individuals ($\sigma_{\delta }$), and scatterplots of the mean of posterior distributions of the overdispersion parameter (θ) regressed against the index of dispersion for each capture–mark–recapture dataset.

Estimates of survival (ϕ) were low relative to truth for CJS models (MSD = −0.047; Coverage = 0.464), but constant (i.e., equivalent to truth) and calibrated (i.e., exhibited appropriate coverage near 0.95) for RD (MSD = −0.003; Coverage = 0.940), ZIP (MSD = −0.002; Coverage = 0.948), and ZIGP (MSD = 0.001; Coverage = 0.948) CMR models (Figure 1; Table 2). Estimates of availability for encounter given previous availability for encounter ($\gamma_{2}|a_{i,t-1}=1$) were slightly underestimated by RD (MSD = −0.020; Coverage = 0.892) and ZIP (MSD = −0.013; Coverage = 0.896) models, but near truth for the ZIGP (MSD = 0.006; Coverage = 0.936) CMR model (Figure 2; Table 2). Estimates of availability for encounter given previous unavailability for encounter ($\gamma_{1}|a_{i,t-1}=0$) were slightly overestimated by RD (MSD = 0.018; Coverage = 0.956), ZIP (MSD = 0.015; Coverage = 0.964), and ZIGP (MSD = 0.019; Coverage = 0.976) CMR models, but coverage was adequate. Estimates of detection probability (*p*) exhibited poor coverage (Figure 3; Table 2) for the RD (MSD = 0.009; Coverage = 0.832) CMR model. Estimates of the average number of reencounters per individual (ϵ) were overestimated with poor coverage with the ZIP (MSD = 0.078; Coverage = 0.764) CMR model, and near truth with the ZIGP (MSD = 0.002; Coverage = 0.928) CMR model. The simulated individual heterogeneity in encounter probability ($\sigma_{\delta }$) in the data was positively correlated with dispersion in the count data (*D*; Figure 4). The overdispersion parameter (θ) in the ZIGP model accounted for some of this overdispersion (Figure 4), improving coverage and constancy for ZIGP models relative to other model types. ZIP and ZIGP models were computationally less expensive than RD models (Figure 4) to sample the same number of iterations.

## 4 DISCUSSION

We demonstrate that CMR models parameterized with zero‐inflated count distributions can function much like robust design CMR models. Estimates of survival probability from RD, ZIP, and ZIGP models were centered around truth, while estimates of survival from the CJS model were consistently low relative to truth. Further, the use of these model types may allow for improved estimation of heterogeneity in encounter probability among individuals and improve computational efficiency (Figure 4). We see substantial utility for these parameterizations in a variety of scenarios. For instance, non‐breeding resights of individuals at wintering or stopover sites may provide an excellent system to model the total number of encounters rather than simple detection/non‐detection data. Further, existing and emerging data types such as camera traps, PIT tags, and automated telemetry may provide large number of detections in discrete time blocks, providing excellent data for the models we describe in this paper.

As we demonstrate, this approach may be particularly useful when unobservable states exist, as counts of reencounters allow for the estimation of a zero‐inflation parameter (i.e., availability for detection), which may be biologically analogous to breeding probability or presence at a stopover or wintering site. Count parameterizations might also be used to model secondary occasions within a robust design model; one or more secondary occasions may be estimated from some count distribution and others from a more typical Bernoulli distribution. The inherent flexibility of programs such as JAGS (Plummer, 2003), NIMBLE (de Valpine et al., 2017), and Stan (Carpenter et al., 2017), and ample literature on capture–reencounter parameterizations should lead to a wide array of extensions of these model types, and their incorporation into joint likelihood models, such as integrated population models (Schaub & Kéry, 2021).

Critically, the use of these model types also has advantages for estimating heterogeneity in detection probability among individuals that are observable, as some individuals may be seen more often than others. Estimating heterogeneity in probabilities from a small number of Bernoulli trials can be challenging (Fay et al., 2022). Summarizing mark–reencounter data as counts of encounters may provide additional information for estimating latent heterogeneity among individuals or estimating mixtures (e.g., Pledger et al., 2003). For example, rather than the heterogeneity parameterization explored in this paper, one might specify a mixture distribution for the number of encounters per individual. Individual covariates can be incorporated simply by modeling the expected number of encounters with a log‐link function. We anticipate a variety of other parameterizations might be useful as well (Table 1) and that simulation work may reveal more effective parameterizations than those described herein. For instance, recent research has demonstrated that a count‐based observation likelihood can be useful for helping to address “false‐positives” in reencounter data (Rakhimberdiev et al., 2022). Thus, we suggest that continued extension of these methods may have broad utility moving forward for capture–reencounter modeling.

**TABLE 1:** Potential parameterizations for zero‐inflated count distribution‐based capture–reencounter models, where $c_{i,t}$ is the number of encounters of individual *i* during occasion *t*, $a_{i,t}$ is an individual's availability for encounter ($a_{i,t}=1$ indicates available; $a_{i,t}=0$ indicates unavailable), and ϵ is the number of expected encounters of an individual.

Parameterization	Model and priors
1. Poisson	$c_{\mathrm{it}}∼\text{Poisson}\left(a_{\mathrm{it}}\times \epsilon \right)$ $\epsilon ∼\text{gamma}\left(1,1\right)$
2. Gamma‐Poisson with individual heterogeneity	$c_{\mathrm{it}}∼\text{Poisson}\left(a_{\mathrm{it}}\times \epsilon \times h_{i}\right)$ $\epsilon ∼\text{gamma}\left(1,1\right)$ $h_{i}∼\text{gamma}\left(\theta ,\theta \right)$ $\theta ∼\text{uniform}\left(0,250\right)$
3. Poisson with two categorical mixtures ($\pi_{i}$)	$c_{\mathrm{it}}∼\text{Poisson}\left(a_{\mathrm{it}}\times \epsilon_{\pi_{i}}\right)$ $\epsilon ∼\text{gamma}\left(1,1\right)$ $\pi_{i}∼\text{categorical}\left(\theta ,1-\theta \right)$ $\theta ∼\text{beta}\left(1,1\right)$
4. Alternative Gamma–Poisson with individual heterogeneity ($\epsilon_{i}$)	$c_{\mathrm{it}}∼\text{Poisson}\left(a_{\mathrm{it}}\times \epsilon_{i}\right)$ $\epsilon_{i}∼\text{gamma}\left(\alpha ,\beta \right)$ $\alpha ∼\text{gamma}\left(1,1\right)$ $\beta ∼\text{gamma}\left(1,1\right)$
5. Lognormal with individual covariates (**X**) and heterogeneity (σ)	$c_{\mathrm{it}}∼\text{Poisson}\left(a_{\mathrm{it}}\times \epsilon_{i}\right)$ $\epsilon_{i}∼\text{lognormal}\left(\beta X,\sigma^{2}\right)$ $\beta ∼\text{normal}\left(0,10\right)$ $\sigma ∼\text{gamma}\left(1,1\right)$

*Note*: We explicitly test parameterizations 1 and 2 in this paper. Parameterization 3 allows for mixtures in encounter probability, where θ is the proportion of individuals in group one, and π is an categorical variable defining the mixture of each individual. Parameterization 4 is similar to parameterization 2, but with a slightly different model for each individual's encounter probability with shape (α) and rate (β) hyperpriors. Finally, parameterization 5 allows for the inclusion of individual covariates (X), associated regression parameters (β), and individual heterogeneity (σ). Please note that a much larger number of potential parameterizations exists, and see Pledger et al. (2003), Greene (2008), Lynch et al. (2014), Kéry and Royle (2015), and McClintock et al. (2009, 2019) for further reading.

**TABLE 2:** Mean difference between the medians of the posterior distributions and truth and parameter coverage (in parentheses) for estimates of apparent survival (ϕ), availability for encounter given $a_{i,t-1}=0$ ($\gamma_{1}$), availability for encounter given $a_{i,t-1}=1$ ($\gamma_{2}$), primary occasion detection probability (*p* [CJS] or p* [RD]), and the expected number of encounters per individual (ϵ) from 250 simulated capture–mark–recapture datasets analyzed using Cormack–Jolly–Seber (CJS; Cormack, 1964; Jolly, 1965; Seber, 1965), robust design (RD; Kendall et al., 1997), zero‐inflated Poisson (ZIP; this study), and zero‐inflated Gamma‐Poisson (ZIGP; this study) capture–recapture models.

Parameter	CJS	RD	ZIP	ZIGP
ϕ	−0.047 (0.464)	−0.003 (0.940)	−0.002 (0.948)	0.001 (0.948)
$\gamma_{1}$	–	0.018 (0.956)	0.015 (0.964)	0.019 (0.976)
$\gamma_{2}$	–	−0.020 (0.892)	−0.013 (0.896)	0.006 (0.936)
*p* (CJS) or p* (RD)	−0.306 (0.004)	0.010 (0.832)	–	–
ϵ	–	–	0.078 (0.764)	0.002 (0.928)

As with the use of any model, violations of model assumptions will lead to inaccurate parameter estimates. We caution against the use of these models when encounters are conditional on previous encounters within a season (i.e., trap happiness). As a particularly problematic example, if the nest of a marked animal is discovered and the animal is then observed repeatedly while visiting the nest, this would serve as an additional type of zero‐inflation (i.e., nesting in the study area is a Bernoulli trial, the discovery of the nest is a Bernoulli trial, and the subsequent visits are a product of study design and nest monitoring protocols, not a random encounter process). We expect that other types of heterogeneity are common in CMR data. For example, the number of encounters might be right truncated if observers cease recording reencounters of individuals that have already been encountered multiple times. Thus, we strongly encourage careful thought about how previous monitoring protocols might affect the distribution of encounters of each individual when applying these models to data and discourage using this approach without explicit information about monitoring protocols.

The use of the Poisson distribution requires the assumption that the mean and the variance are equal. When the encounter data are under or overdispersed, this can lead to respective under or overestimation of the expected number of encounters per individual. Similarly, the probability of availability for encounter will be over or underestimated given under or overdispersion of the encounter data (Figure 4). While overdispersion can be modeled simply using gamma‐Poisson mixture (demonstrated herein) or negative binomial distributions (Table 1), underdispersion requires the use of more complex distributions such as the Conway–Maxwell–Poisson (Conway & Maxwell, 1962; Lynch et al., 2014). We suggest that additional simulation work is required to fully understand the benefits and costs associated with using alternative distributions. Notably, while the authors have not yet developed goodness‐of‐fit tests for these model types, the use of these parameterizations might simplify goodness‐of‐fit testing for capture–reencounter models due to the use of counts rather than Bernoulli trials.

While we have demonstrated in this paper that count‐based observation parameterizations can be useful for capture–mark–reencounter studies, much remains to be learned. For example, careful thought will be required for developing appropriate priors (e.g., Northrup & Gerber, 2018), and empirical research may reveal unforeseen problems. Future simulation work might assess the impacts of priors on inference, further examine the impacts of over‐ and under‐dispersion, and explore various other capture–recapture parameterizations and count distributions.

## AUTHOR CONTRIBUTIONS

**Thomas V. Riecke:** Conceptualization (lead); writing – original draft (lead); writing – review and editing (equal). **Daniel Gibson:** Conceptualization (equal); writing – review and editing (equal). **James S. Sedinger:** Conceptualization (equal); writing – review and editing (equal). **Michael Schaub:** Conceptualization (equal); writing – review and editing (equal).

## CONFLICT OF INTEREST

The authors have no conflict of interest to declare.

## Supporting information


Appendix S1

## Data Availability

All of the data used in this manuscript were simulated. The R script for simulating these data is attached as Appendix S1.
