# Supplementary material for: Zero‐inflated count distributions for capture–mark–reencounter data
Source: Ecol Evol. 2022 Sep 9;12(9):e9274. doi: 10.1002/ece3.9274 (PMC9463028; doi:10.1002/ece3.9274)
Supplement: Supplementary file 1 — Appendix S1 [file ECE3-12-e9274-s001.docx]

# ~~~~~~~~~~~~~~~~~~~~~~~~~~~~~~~~~~~~~~~~~~~~~~~~~~~~~~~~~~~~~~~~~~~~~~~~~~~

# m-array

# ~~~~~~~~~~~~~~~~~~~~~~~~~~~~~~~~~~~~~~~~~~~~~~~~~~~~~~~~~~~~~~~~~~~~~~~~~~~

marray <- function(CH){

nind <- dim(CH)[1]

n.occasions <- dim(CH)[2]

m.array <- matrix(data = 0, ncol = n.occasions+1, nrow = n.occasions)

# Calculate the number of released individuals at each time period

for (t in 1:n.occasions){

m.array[t,1] <- sum(CH[,t])

}

for (i in 1:nind){

pos <- which(CH[i,]!=0)

g <- length(pos)

for (z in 1:(g-1)){

m.array[pos[z],pos[z+1]] <- m.array[pos[z],pos[z+1]] + 1

} #z

} #i

# Calculate the number of individuals that is never recaptured

for (t in 1:n.occasions){

m.array[t,n.occasions+1] <- m.array[t,1] - sum(m.array[t,2:n.occasions])

}

out <- m.array[1:(n.occasions-1),2:(n.occasions+1)]

return(out)

}

# ~~~~~~~~~~~~~~~~~~~~~~~~~~~~~~~~~~~~~~~~~~~~~~~~~~~~~~~~~~~~~~~~~~~~~~~~~~~

# capture-recapture with a Poisson response

# (total number of re-encounters/re-sights) rather

# than a Bernoulli trial (1 or 0)

# ~~~~~~~~~~~~~~~~~~~~~~~~~~~~~~~~~~~~~~~~~~~~~~~~~~~~~~~~~~~~~~~~~~~~~~~~~~~

library(jagsUI)

n.years <- 10 # study duration

releases <- 25 # releases per year

f <- rep(seq(1,n.years-1), each = releases) # first encounter/marking vector

I <- length(f) # total number of individuals

n.days <- 21

n.sec <- 3

sec.length <- n.days/n.sec

n.sims <- 250

nc <- 3

nt <- 10

ni <- 50000

nb <- 10000

tru.phi <- rep(NA, n.sims)

tru.gamma <- matrix(NA, n.sims, 2)

dispersion <- rep(NA, n.sims)

tru.delta <- rep(NA, n.sims)

tru.sigma <- rep(NA, n.sims)

time <- matrix(NA, n.sims, 4)

phi.est <- array(NA, dim = c(n.sims, 4, 7))

gamma.est <- array(NA, dim = c(n.sims, 4, 7, 2))

epsilon.est <- array(NA, dim = c(n.sims, 4, 7))

p.est <- array(NA, dim = c(n.sims, 4, 7))

theta.est <- matrix(NA, n.sims, 7)

# ~~~~~~~~~~~~~~~~~~~~~~~~~~~~~~~~~~~~~~~~~~~~~~~~~~~~~~~~~~~~~~~~~~~~~~~~~~~~~

# begin for loop

# ~~~~~~~~~~~~~~~~~~~~~~~~~~~~~~~~~~~~~~~~~~~~~~~~~~~~~~~~~~~~~~~~~~~~~~~~~~~~~

for (ii in 1:n.sims){

print(ii)

print(Sys.time())

phi <- rbeta(1, 40, 10) # survival

gamma <- NULL

gamma[1] <- rbeta(1, 10, 20) # available for detection

gamma[2] <- rbeta(1, 20, 10) # available for detection

mu.delta <- rbeta(1, 10, 90) # mean detection

sigma.delta <- rgamma(1, 5, 50)

tau.delta <- 1/(sigma.delta * sigma.delta)

delta <- rbeta(I, mu.delta*tau.delta, (1 - mu.delta) * tau.delta) # mean 0.1

# hist(delta)

p.sec <- 1 - (1 - mu.delta)^(sec.length)

pstar <- 1 - (1 - p.sec)^n.sec

eps <- n.days * mu.delta

# pstar <- 1 - (1 - mu.d)^n.days # same as above

tru.phi[ii] <- phi

tru.gamma[ii,1] <- gamma[1]

tru.gamma[ii,2] <- gamma[2]

tru.delta[ii] <- mu.delta

tru.sigma[ii] <- sigma.delta

# ~~~~~~~~~~~~~~~~~~~~~~~~~~~~~~~~~~~~~~~~~~~~~~~~~~~~~~~~~~~~~~~~~~~~~~~~~~~~~

# simulate data

# ~~~~~~~~~~~~~~~~~~~~~~~~~~~~~~~~~~~~~~~~~~~~~~~~~~~~~~~~~~~~~~~~~~~~~~~~~~~~~

z <- matrix(0, I, n.years)

a <- matrix(0, I, n.years)

y <- array(0, dim = c(I, n.years, n.days))

r <- array(0, dim = c(I, n.years, n.sec))

m <- matrix(0, I, n.years)

c <- matrix(0, I, n.years)

for (i in 1:I){

z[i,f[i]] <- 1 # alive when released

a[i,f[i]] <- 1 # available for detection

y[i,f[i],1:n.days] <- -1 # -1 on initial release

m[i,f[i]] <- 1

r[i,f[i],1:n.sec] <- -1

c[i,f[i]] <- -1

for (t in (f[i]+1):n.years){

z[i,t] <- rbinom(1, z[i,t-1], phi) # latent state

a[i,t] <- rbinom(1, z[i,t], gamma[a[i,t-1] + 1]) # Markovian availability for detection

for (j in 1:n.days){

y[i,t,j] <- rbinom(1, a[i,t], delta[i]) # if alive, a mean and variance of 3 resights per individual

}

# robust design data

for (k in 1:n.sec){

if (sum(y[i, t, (sec.length*k - (sec.length - 1)):(sec.length*k)]) >= 1){

r[i,t,k] <- 1

}

}

if (sum(y[i,t,1:n.days]) >= 1){

m[i,t] <- 1

}

c[i,t] <- sum(y[i,t,1:n.days])

}

}

dispersion[ii] <- var(c[a == 1 & c != -1])/mean(c[a == 1 & c != -1])

# ~~~~~~~~~~~~~~~~~~~~~~~~~~~~~~~~~~~~~~~~~~~~~~~~~~~~~~~~~~~~~~~~~~~~~~~~~~~~~

# set up data for JAGS

# ~~~~~~~~~~~~~~~~~~~~~~~~~~~~~~~~~~~~~~~~~~~~~~~~~~~~~~~~~~~~~~~~~~~~~~~~~~~~~

###

### last encounter

###

get.last <- function(x){max(which(x > 0))}

l <- apply(m, 1, get.last)

###

### known alive matrix (provided to JAGS as data)

###

z.dat <- matrix(NA, I, n.years)

for (i in 1:nrow(z.dat)){

z.dat[i,f[i]] <- 1

if (l[i] > f[i]){

z.dat[i, f[i]:l[i]] <- 1

}

}

###

### known available matrix (provided to JAGS as data)

###

a.dat <- m

a.dat[a.dat == 0] <- NA

###

### marray for CJS

###

marr <- marray(m)

rel <- rowSums(marr)

# ~~~~~~~~~~~~~~~~~~~~~~~~~~~~~~~~~~~~~~~~~~~~~~~~~~~~~~~~~~~~~~~~~~~~~~~~~~~~

# 4 model types

# ~~~~~~~~~~~~~~~~~~~~~~~~~~~~~~~~~~~~~~~~~~~~~~~~~~~~~~~~~~~~~~~~~~~~~~~~~~~~

# 1a) CJS m-array

# 1b) CJS latent state

# 2) Robust-design

# 3) zero-inflated Poisson

# 4) zero-inflated Poisson w/heterogeneity

# ~~~~~~~~~~~~~~~~~~~~~~~~~~~~~~~~~~~~~~~~~~~~~~~~~~~~~~~~~~~~~~~~~~~~~~~~~~~~

# ~~~~~~~~~~~~~~~~~~~~~~~~~~~~~~~~~~~~~~~~~~~~~~~~~~~~~~~~~~~~~~~~~~~~~~~~~~~~

# 1a) CJS m-array

# ~~~~~~~~~~~~~~~~~~~~~~~~~~~~~~~~~~~~~~~~~~~~~~~~~~~~~~~~~~~~~~~~~~~~~~~~~~~~

# sink("cjs_marr.jags")

# cat("

# model {

# phi ~ dbeta(1,1)

# p ~ dbeta(1,1)

# for (t in 1:(n.years-1)){

# marr[t, 1:n.years] ~ dmulti(pr[t,1:n.years], rel[t])

# pr[t,t] <- phi * p

# pr[t,n.years] <- 1 - sum(pr[t,1:(n.years-1)])

# }

# for (t in 1:(n.years-2)){

# for (j in (t+1):(n.years-1)){

# pr[t,j] <- pow(phi, (j-t+1)) * pow(1 - p, (j-t)) * p

# }

# }

# for (t in 2:(n.years-1)){

# for (j in 1:(t-1)){

# pr[t,j] <- 0

# }

# }

#}

#",fill = TRUE)

#sink()

# jags.data <- list(n.years = n.years,

# I = I, f = f, marr = marr, rel = rowSums(marr))

# inits <- function(){list(phi = 0.8)}

# parameters <- c('phi', 'p')

# run time: 50k ~ 2s

# Sys.time()

# cjs <- jags(jags.data, inits, parameters, "cjs_marr.jags",

# n.chains = nc, n.thin = nt, n.iter = ni, n.burnin = nb,

# parallel = T)

# Sys.time()

# print(cjs, digits = 3)

# ~~~~~~~~~~~~~~~~~~~~~~~~~~~~~~~~~~~~~~~~~~~~~~~~~~~~~~~~~~~~~~~~~~~~~~~~~~~~

# 1b) CJS latent state

# ~~~~~~~~~~~~~~~~~~~~~~~~~~~~~~~~~~~~~~~~~~~~~~~~~~~~~~~~~~~~~~~~~~~~~~~~~~~~

sink("cjs_latent.jags")

cat("

model {

phi ~ dbeta(1,1)

p ~ dbeta(1,1)

for (i in 1:I){

for (t in (f[i]+1):n.years){

z[i,t] ~ dbern(phi * z[i,t-1])

m[i,t] ~ dbern(z[i,t] * p)

}

}

}

",fill = TRUE)

sink()

jags.data <- list(n.years = n.years, z = z.dat,

I = I, f = f, m = m)

inits <- function(){list(phi = phi)}

parameters <- c('phi', 'p')

# run time: 50k ~ 2m

Sys.time()

cjs <- jags(jags.data, inits, parameters, "cjs_latent.jags",

n.chains = nc, n.thin = nt, n.iter = ni, n.burnin = nb,

parallel = T)

Sys.time()

# print(cjs, digits = 3)

# ~~~~~~~~~~~~~~~~~~~~~~~~~~~~~~~~~~~~~~~~~~~~~~~~~~~~~~~~~~~~~~~~~~~~~~~~~~~~

# 2) robust design

# ~~~~~~~~~~~~~~~~~~~~~~~~~~~~~~~~~~~~~~~~~~~~~~~~~~~~~~~~~~~~~~~~~~~~~~~~~~~~

sink("rd.jags")

cat("

model {

phi ~ dbeta(1,1)

p ~ dbeta(1,1)

gamma[1] ~ dbeta(1,1)

gamma[2] ~ dbeta(1,1)

pstar <- 1 - (1 - p)^(n.sec)

for (i in 1:I){

# robust design capture-recapture likelihood

for (t in (f[i]+1):n.years){

z[i,t] ~ dbern(phi * z[i,t-1])

a[i,t] ~ dbern(z[i,t] * gamma[a[i,t-1]+1])

for (j in 1:n.sec){

r[i,t,j] ~ dbern(a[i,t] * p)

}

}

}

}

",fill = TRUE)

sink()

jags.data <- list(z = z.dat, a = a.dat, n.years = n.years,

I = I, f = f, r = r, n.sec = n.sec)

inits <- function(){list(phi = phi, gamma = gamma)}

parameters <- c('phi', 'p', 'gamma', 'pstar')

# run time: 50k ~ 4 minutes

Sys.time()

rd <- jags(jags.data, inits, parameters, "rd.jags",

n.chains = nc, n.thin = nt, n.iter = ni, n.burnin = nb,

parallel = T)

Sys.time()

# print(rd, digits = 3)

# ~~~~~~~~~~~~~~~~~~~~~~~~~~~~~~~~~~~~~~~~~~~~~~~~~~~~~~~~~~~~~~~~~~~~~~~~~~~~

# 3) Poisson

# ~~~~~~~~~~~~~~~~~~~~~~~~~~~~~~~~~~~~~~~~~~~~~~~~~~~~~~~~~~~~~~~~~~~~~~~~~~~~

sink("zip_cmr.jags")

cat("

model {

phi ~ dbeta(1,1)

gamma[1] ~ dbeta(1,1)

gamma[2] ~ dbeta(1,1)

epsilon ~ dgamma(1,1)

for (i in 1:I){

for (t in (f[i]+1):n.years){

z[i,t] ~ dbern(phi * z[i,t-1])

a[i,t] ~ dbern(z[i,t] * gamma[a[i,t-1]+1])

c[i,t] ~ dpois(a[i,t] * epsilon)

}

}

}

",fill = TRUE)

sink()

# Data

jags.data <- list(z = z.dat, a = a.dat, n.years = n.years,

I = I, f = f, c = c)

# Initial values

inits <- function(){list(phi = phi, gamma = gamma)}

# Parameters

parameters <- c('phi', 'epsilon', 'gamma')

Sys.time()

zip <- jags(jags.data, inits, parameters, "zip_cmr.jags",

n.chains = nc, n.thin = nt, n.iter = ni, n.burnin = nb,

parallel = T)

Sys.time()

# print(zip, digits = 3)

# ~~~~~~~~~~~~~~~~~~~~~~~~~~~~~~~~~~~~~~~~~~~~~~~~~~~~~~~~~~~~~~~~~~~~~~~~~~~~

# 4) Poisson w/ individual heterogeneity

# ~~~~~~~~~~~~~~~~~~~~~~~~~~~~~~~~~~~~~~~~~~~~~~~~~~~~~~~~~~~~~~~~~~~~~~~~~~~~

sink("ziph_cmr.jags")

cat("

model {

phi ~ dbeta(1,1)

gamma[1] ~ dbeta(1,1)

gamma[2] ~ dbeta(1,1)

epsilon ~ dgamma(1,1)

theta ~ dunif(0,250)

for (i in 1:I){

h[i] ~ dgamma(theta, theta)

for (t in (f[i]+1):n.years){

z[i,t] ~ dbern(phi * z[i,t-1])

a[i,t] ~ dbern(z[i,t] * gamma[a[i,t-1]+1])

c[i,t] ~ dpois(a[i,t] * epsilon * h[i])

}

}

}

",fill = TRUE)

sink()

jags.data <- list(z = z.dat,

a = a.dat,

n.years = n.years,

I = I, f = f, c = c)

inits <- function(){list(phi = phi, gamma = gamma)}

parameters <- c('phi', 'epsilon', 'gamma', 'theta')

Sys.time()

ziph <- jags(jags.data, inits, parameters, "ziph_cmr.jags",

n.chains = nc, n.thin = nt, n.iter = ni, n.burnin = nb,

parallel = T)

Sys.time()

# print(ziph, digits = 3)

# survival probability

phi.est[ii, 1, 1:7] <- c(cjs$mean$phi, cjs$sd$phi, cjs$q2.5$phi, cjs$q50$phi, cjs$q97.5$phi, cjs$Rhat$phi, cjs$n.eff$phi)

phi.est[ii, 2, 1:7] <- c(rd$mean$phi, rd$sd$phi, rd$q2.5$phi, rd$q50$phi, rd$q97.5$phi, rd$Rhat$phi, rd$n.eff$phi)

phi.est[ii, 3, 1:7] <- c(zip$mean$phi, zip$sd$phi, zip$q2.5$phi, zip$q50$phi, zip$q97.5$phi, zip$Rhat$phi, zip$n.eff$phi)

phi.est[ii, 4, 1:7] <- c(ziph$mean$phi, ziph$sd$phi, ziph$q2.5$phi, ziph$q50$phi, ziph$q97.5$phi, ziph$Rhat$phi, ziph$n.eff$phi)

# available for detection

gamma.est[ii, 2, 1:7, 1] <- c(rd$mean$gamma[1], rd$sd$gamma[1], rd$q2.5$gamma[1], rd$q50$gamma[1], rd$q97.5$gamma[1], rd$Rhat$gamma[1], rd$n.eff$gamma[1])

gamma.est[ii, 3, 1:7, 1] <- c(zip$mean$gamma[1], zip$sd$gamma[1], zip$q2.5$gamma[1], zip$q50$gamma[1], zip$q97.5$gamma[1], zip$Rhat$gamma[1], zip$n.eff$gamma[1])

gamma.est[ii, 4, 1:7, 1] <- c(ziph$mean$gamma[1], ziph$sd$gamma[1], ziph$q2.5$gamma[1], ziph$q50$gamma[1], ziph$q97.5$gamma[1], ziph$Rhat$gamma[1], ziph$n.eff$gamma[1])

gamma.est[ii, 2, 1:7, 2] <- c(rd$mean$gamma[2], rd$sd$gamma[2], rd$q2.5$gamma[2], rd$q50$gamma[2], rd$q97.5$gamma[2], rd$Rhat$gamma[2], rd$n.eff$gamma[2])

gamma.est[ii, 3, 1:7, 2] <- c(zip$mean$gamma[2], zip$sd$gamma[2], zip$q2.5$gamma[2], zip$q50$gamma[2], zip$q97.5$gamma[2], zip$Rhat$gamma[2], zip$n.eff$gamma[2])

gamma.est[ii, 4, 1:7, 2] <- c(ziph$mean$gamma[2], ziph$sd$gamma[2], ziph$q2.5$gamma[2], ziph$q50$gamma[2], ziph$q97.5$gamma[2], ziph$Rhat$gamma[2], ziph$n.eff$gamma[2])

# detection probability

p.est[ii, 1, 1:7] <- c(cjs$mean$p, cjs$sd$p, cjs$q2.5$p, cjs$q50$p, cjs$q97.5$p, cjs$Rhat$p, cjs$n.eff$p)

p.est[ii, 2, 1:7] <- c(rd$mean$pstar, rd$sd$pstar, rd$q2.5$pstar, rd$q50$pstar, rd$q97.5$pstar, rd$Rhat$pstar, rd$n.eff$pstar)

# encounters per individual

epsilon.est[ii, 3, 1:7] <- c(zip$mean$epsilon, zip$sd$epsilon, zip$q2.5$epsilon, zip$q50$epsilon, zip$q97.5$epsilon, zip$Rhat$epsilon, zip$n.eff$epsilon)

epsilon.est[ii, 4, 1:7] <- c(ziph$mean$epsilon, ziph$sd$epsilon, ziph$q2.5$epsilon, ziph$q50$epsilon, ziph$q97.5$epsilon, ziph$Rhat$epsilon, ziph$n.eff$epsilon)

theta.est[ii, 1:7] <- c(ziph$mean$theta, ziph$sd$theta, ziph$q2.5$theta, ziph$q50$theta, ziph$q97.5$theta, ziph$Rhat$theta, ziph$n.eff$theta)

time[ii,] <- c(cjs$mcmc.info$elapsed.mins, rd$mcmc.info$elapsed.mins,

zip$mcmc.info$elapsed.mins, ziph$mcmc.info$elapsed.mins)

print(Sys.time())

}

# ~~~~~~~~~~~~~~~~~~~~~~~~~~~~~~~~~~~~~~~~~~~~~~~~~~~~~~~~~~~~~~~~~~~~~~~~~~~~~~

# end script

# ~~~~~~~~~~~~~~~~~~~~~~~~~~~~~~~~~~~~~~~~~~~~~~~~~~~~~~~~~~~~~~~~~~~~~~~~~~~~~~
